# Carriage of Ser217Leu and Ala541Thr Variants of ELAC2 Gene and Risk Factors in Patients with Prostate Cancer in Burkina Faso

**DOI:** 10.1155/2022/3610089

**Published:** 2022-12-17

**Authors:** Aïda Djé Djénèba Traoré, Bienvenu Désiré Ky, Lassina Traoré, Théodora M. Zohoncon, Abdou Azaque Zouré, Albert Théophane Yonli, Herman Karim Sombié, Pegdwendé Abel Sorgho, Bapio Valery Jean Télesphore Elvira Bazié, Sessi Frida Appoline Tovo, Essonan Kadanga, Bélélé Siméon Bakyono, Kalifou Traore, Teega-Wendé Clarisse Ouédraogo, Florencia W. Djigma, Jacques Simpore

**Affiliations:** ^1^Laboratory of Molecular and Genetic Biology (LABIOGENE), Joseph KI-ZERBO University, 03 BP 7021, Ouagadougou 03, Burkina Faso; ^2^Pietro Annigoni Biomolecular Research Center (CERBA), 01 BP 364, Ouagadougou 01, Burkina Faso; ^3^Urology Department CHU Yalgado Ouedraogo UFR SDS, Joseph KI ZERBO University, 03 BP 7021 Ouagadougou 03, Ouagadougou, Burkina Faso; ^4^Faculty of Medicine, Saint Thomas Aquinas University (USTA), 06 BP 10212, Ouagadougou 06, Burkina Faso; ^5^Biomedical Research Laboratory (LaReBio) Biomedical and Public Health Department, Institute for Research in Health Sciences (IRSS/CNRST), 03 BP 7192, Ouaga03, Burkina Faso; ^6^Institute for Research in Applied Sciences (IRSAT/CNRST), 03 BP 7192, Ouaga03, Burkina Faso

## Abstract

**Background:**

Genetic factors are one of the significant contributors to prostate cancer (PCa) development, and hereditary prostate cancer 2 (HPC2) locus gene ELAC2 is considered a PCa susceptibility region. The HPC2/ELAC2 gene has been identified by linkage analysis in familial prostate cancer patients in the United States but has never been studied in Burkina Faso. The objective of the present study was to analyze the carriage of the C650T (Ser217Leu) and G1621A (Ala541Thr) mutations of the ELAC2 gene and the risk factors in prostate cancer patients in Burkina Faso.

**Methods:**

This case-control study included 76 participants, including 38 histologically confirmed prostate cancer cases and 38 healthy controls without prostate abnormalities. PCR combined with restriction fragment length polymorphism (RFLP) was used to characterize the genotypes of the Ser217Leu and Ala541Thr polymorphisms of the ELAC2 gene. The correlations between the different genotypes and risk factors for prostate cancer were investigated.

**Results:**

The C650T mutation was present in 44.73% of prostate cancer cases and 47.37% of controls. The G1621A mutation was present in 26.32% of prostate cancer cases and 15.79% of controls. We did not detect an association between prostate cancer risk and the Ser217Leu (*p*=0.972) and Ala541Thr (*p*=0.267) variants of the ELAC2 gene. Also, the two ELAC2 SNPs did not correlate with clinical stage, prostate-specific antigen (PSA) level at diagnosis, or the Gleason score on biopsies. However, we found that 100% of homozygous carriers of the T650 mutation have an A1621 mutation (*p* ≤ 0.001).

**Conclusion:**

Ser217Leu and Ala541Thr polymorphisms of ELAC2, considered alone or in combination, are not associated with prostate cancer risk.

## 1. Introduction

Worldwide, approximately, 19.3 million new cancer cases and nearly 10 million cancer deaths occurred in 2020 [1]. Prostate cancer (PCa) is the second most common malignancy (after lung cancer) [1], with 1,414,259 new cases and 375,304 deaths (3.8% of all cancer deaths in men) in 2020. In Burkina Faso, it is the most common malignant tumor in men. In the same year, PCa accounted for 8.30% of incident cancer cases (997 cases) and 7.00% of cancer deaths (608) [2]. Although the exact etiology of prostate cancer remains elusive, well-established risk factors include advanced age, ethnicity, and family history [3]. Populations of African ancestry, such as African Americans, Caribbeans, and blacks in Europe, had the highest incidences, earlier disease, and more aggressive form compared with other racial and ethnic groups [4]. Men of African ancestry are estimated to have a relative risk of 9.7 versus 3.9 for Caucasians and 1.6 for Asians, when two or more first-degree relatives have PCa [5]. Regarding family history, more than 20% of patients with prostate cancer report a family history [6]. This is partly due to shared genes but also due to a similar pattern of exposure to certain environmental carcinogens and common lifestyles [3, 6]. The prostate-specific antigen (PSA) test and digital rectal exam (DRE) are currently the main methods of noninvasive prostate cancer screening [7]. These tests are not specific because some of the PSA levels tested may result in false positives due to infection or hyperplasia. With the ultimate goal of developing new, more accurate, and beneficial biomarkers in the detection, prevention, and treatment of this disease, several genome-wide linkage and association studies (GWAS) have been conducted to elucidate the molecular mechanisms involved in the genesis and progression of prostate cancer [8]. Tavtigian et al. in 2001 [9] identified the HPC2/ELAC2 gene on chromosome 17p12 as a prostate cancer susceptibility gene in large, high-risk pedigrees in Utah. Subsequently, several other loci associated with inherited forms of PCa were identified, including HPC1 (1q24-25), HPC20 (20q13), HPCX (Xq27-28), PG1 (prostate cancer susceptibility gene 1, 8p22-23), and CAPB (1p36) [10]. Two additional inherited PCa susceptibility genes have been identified at two of these loci. These are the RNASEL (2'-5'-oligoadenylate-dependent ribonuclease L) and MSR1 (macrophage scavenger receptor 1) genes [11–13]. However, mutations in these different genes have low to moderate penetrance.

The ELAC2 gene located at position 17p12, with a size of 26.454 kb, having 25 exons, encodes a metal-dependent hydrolase of 826 amino acids (of 92 kilo-Dalton) potentially involved in the repair of DNA interstrand cross-linking and mRNA editing [14, 15]. Sequence analysis of HPC2/ELAC2 identified four sequence variants, including a rare frameshift and three missense changes, two of which were common in the study populations [9, 16–18]. These two missense mutations C650T and G1621A result in a change of the amino acid serine to leucine at position 217 and a change of alanine to threonine at position 541, respectively. However, conflicting results regarding the association of ELAC2 gene polymorphisms and the occurrence of PCa have been obtained in different populations around the world, testifying to the genetic complexity and heterogeneity of prostate cancer susceptibility [17, 19–25]. In West Africa, and specifically in Burkina Faso, several studies on the epidemiological and therapeutic aspects of PCa have been carried out, but only a few studies on the genetic and molecular aspects. It is with this in mind that this preliminary study was conducted to determine the involvement of the Ser217Leu (C650T) and Ala541Thr (G1621A) variants of the ELAC2 gene in prostate cancer in the Burkinabe population. This could provide additional information that could potentially be exploited for early screening and diagnoses of high-risk individuals for early therapeutic intervention or ease of management.

## 2. Materials and Methods

### 2.1. Design of Study

This is a case-control study that was conducted from September, 2019 to January, 2021. It included men, regardless of ethnicity, at least 60 years old (for controls), with a positive (cases) or negative (controls) histological diagnosis of prostate adenocarcinoma who consented to participate in the study. This study involved a population of all professions and social categories. Seventy-six (76) men were selected, including 38 cases and 38 controls. All patients were followed at the urology department of the Saint Camille Hospital in Ouagadougou (HOSCO) or the Nina clinic in Ouagadougou. Biomolecular analyses were performed at the Laboratory of Molecular and Genetic Biology (LABIOGENE) of the Joseph KI-ZERBO University in Ouagadougou and the Pietro Annigoni Biomolecular Research Center (CERBA) in Ouagadougou (Burkina Faso).

### 2.2. Sample Collection

After consent was obtained from the patients (cases and controls), a questionnaire was distributed to collect sociodemographic and clinical data from the participants. Then, for each consenting participant, venous blood (5 ml) was collected in tubes impregnated with ethylenediaminetetraacetic acid (EDTA). After centrifugation at 3500 g for 15 minutes, the plasma and pellets were collected in cryotubes and stored at −20°C at CERBA, pending nucleic acid extraction.

### 2.3. Quantification of Total PSA

The sera were used for the determination of total PSA in the HOSCO laboratory on the Cobas® 6000 Analyzer (Basel, Switzerland) using the Elecsys® Total PSA reagent (Roche, Basel, Switzerland) employing the electrochemiluminescence (ECLIA) method.

### 2.4. Genotyping

Genomic DNA was extracted from the pellet using the “Rapid Salting Out” technique, described by Miller et al. in 1988 [26]. The two single-nucleotide polymorphisms (SNPs) in the HPC2/ELAC2 gene were genotyped for all subjects using PCR-RFLP, as described by Xu et al. [20]. In brief, conventional PCR was performed for the amplification of regions carrying the Ser217Leu and Ala541Thr polymorphisms of ELAC2 on the Gene Amp® PCR system 9700 in a 20 *μ*L reaction volume, containing 4 *μ*L of 5X FIREPOL® Master Mix (Solis Biodyne, Riia, Estonia), 0.5 *μ*L (0.2 *μ*M) of two primers [20] (Table 1), 10 *μ*L of reagent grade water and 5 *μ*L of DNA (10 ng/*μ*L). The PCR program used was 94°C for 5 min, 35 cycles consisting of 94°C for 30 s, 55°C for 30 s, and 72°C for 30 s, followed by a final extension of 72°C for 5 min for the Ser217Leu variant. Amplicons of 276 bp were confirmed on 2% agarose gels stained with ethidium bromide. For the Ala541Thr polymorphism, PCR conditions were the same as for Ser217Leu, except that the hybridization temperature was 57°C. For this variant, a 495 bp fragment was amplified.

**Figure 1:**
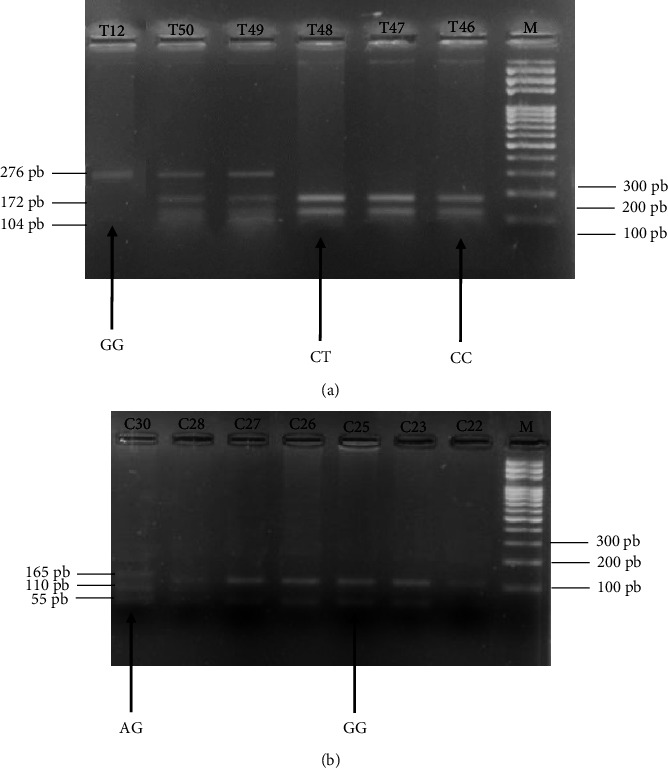
Photographs of agarose gels showing *PCR-RFLP* profiles of Ser217Leu and Ala541Thr polymorphisms of the *ELAC2* gene. (a) Ser217Leu polymorphism. PCR-amplified DNA fragments containing the Ser217Leu polymorphisms were digested with Taq α I and then subjected to 2% agarose gel electrophoresis. The TT mutant genotype generates one DNA fragment of 276 bp, the CT genotype generates three DNA fragments of sizes 276 bp, 172 bp, and 104 bp, and the CC wild type generates two DNA fragments of sizes 172 bp and 104 bp. (b) Ala541Thr polymorphism. PCR-amplified DNA fragments containing the Ala541Thr polymorphisms were digested with Fnu4HI and then subjected to 2% agarose gel electrophoresis. The AA mutant genotype (not found in the population), reportedly generated DNA fragments of 165 bp, the GA genotype generated three DNA fragments of sizes 165 bp, 110 bp, and 55 bp, and the GG wild type generated DNA fragments of sizes 110 bp and 55 bp.

The resulting PCR products were subjected to enzymatic digestion with the enzyme Taq *α* I, 20,000 units/mL (New England Biolabs, Paris, France) at 65°C for 3 hours for the Ser217Leu polymorphism (with one restriction site on the amplified fragment) and Fnu4HI, 20,000 units/mL (New England Biolabs, Paris, France) at 37°C for 3 hours for the Ala541Thr variant (with three restriction sites on the amplified fragment). The total reaction medium of 25 *μ*L included 5 *μ*L of 1X enzyme buffer, 0.5 *μ*L of the enzyme, 14.5 *μ*L of sterile PCR water, and 5 *μ*L of PCR product. Digestion products were subjected to 2% agarose gel electrophoresis for 45 minutes and visualized under UV light at 132 nm using an image analyzer VILBER (Baden-Württemberg, Germany). The PCR-RFLP patterns for the Ser21Leu and Ala541Thr polymorphisms are indicated in Figure 1.

### 2.5. Statistical Analysis

Data were entered into Excel 2016 spreadsheet and analyzed using Stata version 13.0 (https://www.Stata.com) and IBM® SPSS software (https://www.IBM.com). Association of categorical variables was performed using the chi-square test and considered significantly different at *p*  <  0.05. Differences in genotype frequencies between cases and controls were tested using standard chi-square tests. Odd ratio (OR) and 95% confidence interval (CI) were calculated by genotype with Epi infoTM 7 software (Center for Disease Control and Prevention, Atlanta, Georgia, USA).

## 3. Result

### 3.1. Sociodemographic Characteristics

Table 2 summarizes the sociodemographic characteristics and behavioral risk factors selected for PCa. The age of the cases ranged from 55 to 84 years (mean age 69.81 ± 8.05 years), while the age of the controls ranged from 60 to 90 years (mean age 69.11 ± 6.46 years), indicating no difference in the mean age of the case and control groups (*p*=0.668). The age distribution at diagnosis showed that 59.46% of the cases were diagnosed between 50 and 70 years of age and 40.54% over 70 years of age. The mean age at diagnosis was 67.46 ± 8.03 years. A total of 63.15% of PCa patients and 50.00% of controls were alcohol users. There was no difference in the fractions of alcohol consumers and non-consumers among the case and control groups (*p*=0.354). Regarding the presence of a family history of PCa, 36.84% of cases answered yes versus 28.95% of controls, indicating a non-statistically significant difference (*p*=0.551).

### 3.2. Patients' Plasma PSA Level and Gleason Score of the Prostate Gland at Diagnosis

The range of patients' diagnostic PSA at diagnosis levels and the Gleason scores of the prostate is shown in Table 3. The majority of cases (81.6%) had a total PSA level at diagnosis greater than 20 ng/mL (range 22.5 ng/mL to 4028.33 ng/mL). The Gleason score of 7 was the most represented (60.5%).

### 3.3. Allelic Frequencies

We first compared the allele frequencies regarding Ser217Leu and Ala541Thr polymorphisms in HPC2/ELAC2 gene in Burkinabe men with PCa and control subjects. Ser217Leu and Ala541Thr polymorphisms were in the Hardy–Weinberg equilibrium in our study population. The differences in the allele frequencies for Ser217Leu polymorphisms (C and T alleles) and Ala541Thr polymorphisms (G and A alleles) are shown in Table 4. There was no significant difference in the frequency of C or T alleles (*p*=1.000) or G and A alleles (*p*=0.289) between the case and control groups (Table 4).

### 3.4. Genotypic Frequencies of the ELAC2 Gene and Association with Prostate Cancer

The differences in genotypic frequencies of the Ser217Leu polymorphisms (C and T alleles) and the Ala541Thr polymorphisms (G and A alleles) are shown in Table 5. The genotypic frequencies of the Ser217Leu variant were 55.27% for the wild-type CC, 39.47% for the heterozygous CT, and 5.26% for the homozygous mutant TT in cases, and 52.63%, 42.11%, and 5.26% in controls, respectively (*p*=0.972). For the Ala541Thr variant, the genotypic frequencies for the wild-type GG, heterozygous GA, and homozygous mutant AA were 73.68%, 26.32%, and 0.0% in patients and 84.21%, 15.79%, and 0.0%, respectively, in controls (*p*=0.267). No correlation between Ser217Leu and Ala541Thr polymorphisms and the occurrence of prostate cancer was obtained. However, for the Ala541Thr polymorphism, the GA genotype was associated with a family history of PCa (OR = 4.57 (1.13–18.47); *p*=0.050). Also, 87.5% of GA genotype carriers had at least one T allele of the Ser217Leu polymorphism.

### 3.5. ELAC2 Genotypes and Gleason Score of the Disease

The differences in the allele frequencies for Ser217Leu polymorphisms (C and T alleles) and Ala541Thr polymorphisms (G and A alleles) are shown in Table 4. There was no significant difference in the frequency of C or T alleles (*p*=1.000) or G and A alleles (*p*=0.293) among the case and control groups (Table 4).

**Figure 2:**
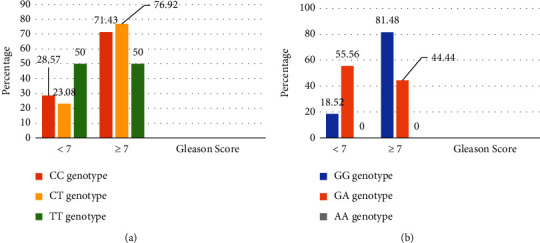
(a) Association between the Gleason score and Ser217Leu mutation. (b) Association between the Gleason score and Ala541Thr mutation.

For the Ser217Leu mutation, 71.43%, 76.92%, and 50.0% of carriers of the CC, CT, and TT genotypes, respectively, had a Gleason score ≥7. For the Ala541Thr mutation, 81.48%, 44.44%, and 0.00% of carriers of the GG, GA, and AA genotypes, respectively, had a Gleason score ≥7 (Figure 2). No significant association was found between these variants and the Gleason score (Ser217Leu: *p*=0.725, Ala541Thr: *p*=0.072).

### 3.6. Associations of Ser217Leu and Ala541Thr Polymorphisms with PSA at Diagnosis

**Figure 3:**
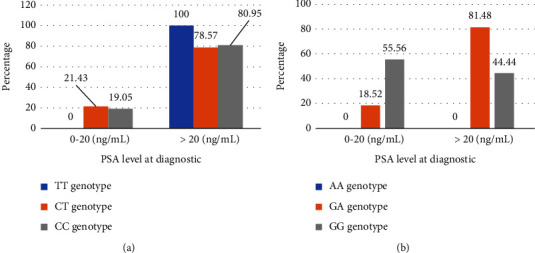
(a) Association between PSA level at diagnosis and Ser217Leu mutation. (b) Association between PSA level at diagnosis and Ala541Thr mutation.

All patients (100%) with the TT mutated genotype of Ser217Leu had a PSA >20 ng/ml. In contrast, carriers of the CC (19.05%) and CT (21.43%) genotypes had PSA levels between 5.38 and 20 ng/ml. For Ala541Thr, 81.48% of GA heterozygous patients had PSA levels above 20 ng/ml. In contrast, the majority (55.56%) of patients with the wild-type GG genotype had the lowest PSA levels at diagnosis (Figure 3). However, no association was found between these mutations and a PSA level at diagnosis greater than 20 ng/ml (Ser217Leu: *p*=0.773; Ala541Th: *p*=0.925).

## 4. Discussion

Many studies have reported that black men are diagnosed with prostate cancer at a younger age [27, 28]. We obtained a mean age of patients at diagnosis of 67.46 ± 8.03 years with an age range of 51 to 81 years, similar to the age range found in Burkina Faso in 2022 and Cameroon (67.8 ± 7.44 years) in 2019 [25, 29]. These results could be attributed to late diagnosis and the low rate of prostate cancer screening programs in Burkina Faso, highlighting the high PSA levels at diagnosis obtained in our study (mean 642.12 ± 1153.42 ng/ml). These levels are almost similar to those obtained (mean PSA of 537 ng/ml et 627.85 ng/ml) in a previous study in Burkina Faso [29, 30]. Regarding the Gleason score, 73.65% of the patients had a Gleason score of 7 or higher. One of the established risk factors for prostate cancer is the presence of a family history. The risk would be increased when there are more than three 1^st^ and/or 2^nd^-degree relatives with PCa [31]. In our study, only 36.84% of patients reported a family history of prostate cancer.

The ELAC2 gene was genotyped in 76 study participants and the frequency of each genotype was determined. For the Ser217Leu polymorphism, the frequencies of the genotypes, CC, CT, and TT were 55.27%, 39.47%, and 5.26% in cases, and 52.63%, 42.11%, and 5.26% in controls, respectively (*p*=0.972). For the Ala541Thr variant, the frequencies of the GG, GA, and AA genotypes were 73.68%, 26.32%, and 0.0% in cases and 84.21%, 15.79%, and 0.0% in controls, respectively (*p*=0.267). The AA mutated genotype was not found in our study population. The HPC2/ELAC2 locus was first discovered as a prostate cancer susceptibility gene through a genetic linkage study by Tavtigian et al. in 2001 [9]. However, most subsequent studies have focused on its role as a common gene with low penetrance [32, 33]. In our study, we did not find a direct correlation between the Ser217Leu (*p*=1.000) and Ala541Thr (*p*=0.293) polymorphisms in the ELAC2 gene and the occurrence of prostate cancer. Our results are similar to those found by Suzuki et al. [23] in Japan, Stanford et al. [17] in the United States, Shea et al. [34] in the Afro-Caribbean population of Tobago, and Meitz et al. [35] in the United Kingdom. In general, the meta-analysis by Xu et al. [21] obtained evidence of an association between the Ser217Leu polymorphism and prostate cancer risk in Caucasians and Asians, but not in Africans. In addition, Xu et al. [21] found that the Ala541Thr polymorphism was associated with an increased risk of prostate cancer in Asians but not in Caucasians and Africans in all genetic models (somatic and inherited). However, several other studies show rather contradictory results regarding the association between Ser217Leu and Ala541Thr variants and prostate cancer risk. Indeed, Yokomizo et al. [19] in Japan found that only the Thr541 allele was associated with an increased risk of PCa; they obtained a significantly higher frequency of this allele in PCa patients (8.4%) compared to the control group (2.1%) (*p*=0.003). In contrast, Robbins et al. [36] found that only the Leu217 allele was significantly associated with prostate cancer risk in the African American population (*p*=0.030). Comparing the frequencies of carriers of both mutations simultaneously in cases and controls, we found that 100% of homozygous carriers of the T650 mutation have an A1621 mutation (*p* ≤ 0.001). Rebbeck et al. [32] found that the A1621 mutation was only observed in men who also carried T650 and that the probability of developing prostate cancer was increased in men who carried the Leu217/Thr541 variants simultaneously (OR = 2.37, 95% CI 1.06–5.29, and *p*=0.040). To determine whether the Ser217Leu and Ala541Thr mutations might be involved in increased disease severity, we compared PSA levels and prostate Gleason scores to genotype frequencies. The result showed that the mutations were not involved in the increased disease severity (*p*  >  0.050).

The differences between our results and those of other studies could be attributed to the difference in sample size on the one hand and on the other hand, to the different ethnic origins and geographical locations of the various populations studied. Indeed, a small sample size lacks statistical power to detect associations. Also, several studies show that there is a correlation between prostate cancer patients and their ethnic background. The allelic frequency of single-nucleotide polymorphisms (SNPs) is also known to differ between races and ethnic groups [23, 37].

### 4.1. Limitations of the Study

The main limitation of our study was the small sample size, not allowing us to conclude on the exact role of the Ser217Leu and Ala541Thr variants of the ELAC2 gene in prostate cancer susceptibility. Second, the mean age at diagnosis of the cases was 67.46 ± 8.03 years, which limits the sample size, as life expectancy was estimated to be 60.1 years in 2019 in Burkina Faso [37].

## 5. Conclusion

Our preliminary study found no direct association between the Ser217Leu and Ala541Thr variants of the HPC2/ELAC2 locus and prostate cancer risk. The patients included in this study had advanced disease. A larger study is needed to better understand the role of different SNPs of ELAC2, other risk factors, and other genes in prostate cancer predisposition, which may benefit early diagnosis of the disease in at-risk individuals and management of the disease.

## Figures and Tables

**Table 1:** Primers for amplification of Ser217Leu and Ala541Thr polymorphisms [20].

Variant	Primers		Size (pb)
*Ser217Leu*	R	5'-CATTCCCATGTATGAACGTCT-3'	276
	F	5'-AGGAAACAGCTATGACCATCTACAAGCATTACAAGGCAGAG-3'	

*Ala541Thr*	F	5'-CCAGCCTTTGTGTAAGTCTAC-3'	495
	R	5'-TCTGGGCAAGTTTGGAAGC-3'	

**Table 2:** Sociodemographic and behavioral characteristics.

Parameters	Cases (*n* = 38) (%)	Controls (*n* = 38) (%)	*p*
Mean age (years)	69.81 ± 8.05	69.11 ± 6.46	*0.668*
*Age at diagnosis (years)*			
50–70	59.46		
71–81	40.54		
Mean age at diagnosis (years)	67.46 ± 8.03		
Consumers of alcohol	63.15	50.00	*0.354*
Presence of PCa family history	36.84	28.95	*0.551*

**Table 3:** Biological characteristics.

Subgroup	Cases (*n* = 38) *n* (%)
*PSA at diagnosis (ng/mL)*	
4.0	0 (0)
4.1–10.0	3 (7.9)
10.1–20.0	4 (10.5)
>20	31 (81.6)
Mean PSA at diagnosis (ng/mL	642.12

*Gleason score*	
6	10 (26.3)
7	23 (60.5)
8	3 (7.89)
9	2 (5.26)

**Table 4:** Allelic frequencies of Ser217Leu and Ala541Thr in cases and controls.

	Allele	Cases (af)	Controls (af)	*p*	OR (95% CI)
Ser217Leu	C	36 (0.75)	36 (0.74)	1.000	0.94 (0.42–2.14)
	T	17 (0.25)	18 (0.26)		

Ala541Thr	G	38 (0.87)	38 (0.92)	0.289	2.0 (0.68–5.88)
	A	12 (0.13)	6 (0.07)		

af = allelic frequency.

**Table 5:** Association between ELAC2 gene genotypes and prostate cancer.

Genotypes		Controls	Cases		PCaWFH vs. controls		PCaFH vs. controls		Cases vs. controls	
Ser217Leu	Ala541Thr		CPS (%)	CPH (%)	OR (95% IC)	*p*	OR (95% IC)	*p*	OR (95% IC)	*p*
CC		20 (52.63)	12 (66.67)	6 (42.86)	Reference					
CT		16 (42.11)	5 (27.78)	7 (50.00)	0.52 (0.15–1.79)	0.374	1.46 (0.41–5.21)	0.747	0.89 (0.35–2.27)	1.000
TT		2 (5.26)	1 (5.55)	1 (7.14)	0.83 (0.06–0.20)	1.000	1.67 (0.13–21.73)	1.000	0.95 (0.12–7.42)	1.000
CT/TT		18 (47.37)	6 (33.33)	8 (57.14)	0.55 (0.17–1.79)	0.393	1.48 (0.43–5.09)	0.755	0.90 (0.36–2.21)	1.000
	GG	32 (84.21)	14 (77.78)	7 (57.14)	Reference					
	GA	6 (15.79)	4 (22.22)	6 (42.86)	1.52 (0.37–6.26)	0.710	4.57 (1.13–18.47)	0.050	1.90 (0.61–5.90)	0.393
	AA	0 (0.00)	0 (0.00)	0 (0.00)	NA	NA	NA	NA	NA	NA
	GA/AA	6 (15.79)	1 (8.33)	2 (25.00)	1.52 (0.37–6.26)	0.710	4.57 (1.13–18.47)	0.050	1.90 (0.61–5.90)	0.393
CC	GG	19 (50.00)	11 (61.11)	6 (46.15)	Reference					
CT/TT	GG	13 (34.21)	3 (16.67)	3 (23.08)	0.39 (0.09–1.71)	0.322	0.73 (0.15–3.46)	1.000	0.58 (0.19–1.72)	0.425
CC	GA/AA	1 (2.63)	1 (5.55)	0 (0.00)	1.72 (0.09–0.15)	1.000	NA	NA	0.95 (0.05-16-26)	1.000
CT/TT	GA/AA	5 (13.16)	3 (16.67)	4 (30.77)	1.04 (0.21–5.19)	1.000	2.53 (0.51–12.59)	0.395	1.71 (0.48–6.03)	0.533

NA: not applicable, PCaFH: prostate cancer with family history, and PCaWFH: prostate cancer without family history.

## Data Availability

All data used to support the findings of this study are available from the corresponding author upon request.

## References

[1] Sung H., Ferlay J., Siegel R. L. (2020). Global Cancer Statistics 2020: GLOBOCAN Estimates of Incidence and Mortality Worldwide for 36 Cancers in 185 Countries. *CA: A Cancer Journal for Clinicians*.

[2] Oms (2020). Cancer Country Profile 2020.

[3] Keng L. N. (2021). The Etiology of Prostate Cancer. *Exon Publications*.

[4] Rebbeck T. R. (2017). Prostate cancer genetics: variation by race, ethnicity, and geography. *Seminars in Radiation Oncology*.

[5] Whittemore A. S., Wu A. H., Kolonel L. N. (1995). Family history and prostate cancer risk in black, white, and Asian men in the United States and Canada. *American Journal of Epidemiology*.

[6] Albright F., Stephenson R. A., Agarwal N. (2015). Prostate cancer risk prediction based on complete prostate cancer family history. *The Prostate*.

[7] Hernández J., Thompson I. M. (2004). Prostate-specific antigen: a review of the validation of the most commonly used cancer biomarker. *Cancer*.

[8] Brandão A., Paulo P., Teixeira M. R. (2020). Hereditary predisposition to prostate cancer: from genetics to clinical implications. *International Journal of Molecular Sciences*.

[9] Tavtigian S. V., Simard J., Teng D. H. F. (2001). A candidate prostate cancer susceptibility gene at chromosome 17p. *Nature Genetics*.

[10] Simard J., Dumont M., Labuda D. (2003). Prostate cancer susceptibility genes: lessons learned and challenges posed. *Endocrine-Related Cancer*.

[11] Rossmanith W., Lightowlers B. (2011). Localization of human RNase Z isoforms: dual nuclear/mitochondrial targeting of the ELAC2 gene product by alternative translation initiation. *PLoS One*.

[12] Rennert H., Zeigler-Johnson C. M., Addya K. (2005). Association of susceptibility alleles in ELAC2/HPC2, RNASEL/HPC1, and MSR1 with prostate cancer severity in European American and african American men. *Cancer Epidemiology, Biomarkers & Prevention*.

[13] Cybulski C., Wokołorczyk D., Jakubowska A. (2007). DNA variation in MSR1, RNASEL and E-cadherin genes and prostate cancer in Poland. *Urologia Internationalis*.

[14] (2017). GeneCards - human genes | gene database | gene search. *Gène ELAC2 (Codage des protéines)*.

[15] Korver W., Guevara C., Chen Y. (2003). The product of the candidate prostate cancer susceptibility gene ELAC2 interacts with the *γ*-tubulin complex. *International Journal of Cancer*.

[16] Zahiri Z., Zahiri F. (2020). A study of Ser217Leu and Ala541Thr polymorphism in the men afflicted with prostate cancer and in the men being suspicious of prostate cancer. *Asian Pacific Journal of Cancer Prevention*.

[17] Stanford J. L., Sabacan L. P., Noonan E. A. (2003). Association of HPC2/ELAC2 polymorphisms with risk of prostate cancer in a population-based study. *Cancer Epidemiology Biomarkers & Prevention*.

[18] İzmirli M., Arikan B., Bayazit Y., Alptekin D. (2011). Polymorphisms of HPC2/ELAC2 and SRD5A2 (5*α*-reductase type II) genes in prostate cancer. *Balkan Journal of Medical Genetics: BJMG*.

[19] Yokomizo A., Koga H., Kinukawa N. (2004). HPC2/ELAC2 polymorphism associated with Japanese sporadic prostate cancer. *The Prostate*.

[20] Xu J., Zheng S. L., Carpten J. D. (2001). Evaluation of linkage and association of HPC2/ELAC2 in patients with familial or sporadic prostate cancer. *The American Journal of Human Genetics*.

[21] Xu B., Tong N., Li J. M., Zhang Z. D., Wu H. f (2010). ELAC2 polymorphisms and prostate cancer risk: a meta-analysis based on 18 case–control studies. *Prostate Cancer and Prostatic Diseases*.

[22] Wu Y. Q., Chen H., Rubin M. A., Wojno K. J., Cooney K. A. (2001). Loss of heterozygosity of the putative prostate cancer susceptibility gene HPC2/ELAC2 is uncommon in sporadic and familial prostate cancer. *Cancer Research*.

[23] Suzuki K., Ohtake N., Nakata S. (2002). Association of HPC2/ELAC2 polymorphism with prostate cancer risk in a Japanese population. *Anticancer Research*.

[24] Severi G., Giles G. G., Southey M. C. (2003). ELAC2/HPC2 polymorphisms, prostate-specific antigen levels, and prostate cancer. *JNCI Journal of the National Cancer Institute*.

[25] Djomkam A. L. Z., Beyeme Sala T., Baari Memba C., Njimoh D. L. (2019). Prevalence of the Ser217Leu variant of the ELAC2 gene and its association with prostate cancer in population of the littoral region of Cameroon. *Prostate Cancer*.

[26] Miller S. A., Dykes D. D., Polesky H. F. (1988). A simple salting out procedure for extracting DNA from human nucleated cells. *Nucleic Acids Research*.

[27] Ravery V., Javerliat I., Toublanc M., Boccon-Gibod L., Delmas V., Boccon-Gibod L. (2000). Features of prostatic cancer in French individuals of African-Caribbean origin. *Progrès en Urologie*.

[28] Freedland S. J., Sutter M. E., Naitoh J., Dorey F., Csathy G. S., Aronson W. J. (2000). Clinical characteristics in black and white men with prostate cancer in an equal access medical center. *Urology*.

[29] Kadanga E., Zouré A. A., Zohoncon T. M. (2022). Carriage of mutations R462Q (rs 486907) and D541E (rs 627928) of the RNASEL gene and risk factors in patients with prostate cancer in Burkina Faso. *BMC Medical Genomics*.

[30] Kabore F. A., Zango B., Sanou A., Yameogo C., Kirakoya B. (2011). Prostate cancer outcome in Burkina Faso. *Infectious Agents and Cancer*.

[31] Rawla P. (2019). Epidemiology of prostate cancer. *World Journal of Oncology*.

[32] Rebbeck T. R., Walker A. H., Zeigler-Johnson C. (2000). Association of HPC2/ELAC2 genotypes and prostate cancer. *The American Journal of Human Genetics*.

[33] Chen Y. C., Giovannucci E., Kraft P., Jhunter D. (2008). Sequence variants of elaC homolog 2 (*Escherichia coli*) (ELAC2) gene and susceptibility to prostate cancer in the Health Professionals Follow-Up Study. *Carcinogenesis*.

[34] Shea P. R., Ferrell R. E., Patrick A. L., Kuller L. H., Bunker C. H. (2002). ELAC2 and prostate cancer risk in Afro-Caribbeans of Tobago. *Human Genetics*.

[35] Meitz J. C., Edwards S. M., Easton D. F. (2002). HPC2/ELAC2 polymorphisms and prostate cancer risk: analysis by age of onset of disease. *British Journal of Cancer*.

[36] Robbins C. M., Hernandez W., Ahaghotu C. (2008). Association of HPC2/ELAC2 and RNASEL non-synonymous variants with prostate cancer risk in African American familial and sporadic cases. *The Prostate*.

[37] INdlSedl D. (2019). Cinquième Recensement Général de la Population et de l’Habitation du Burkina Faso. *Résultats Préliminaires*.

